# Viricidal Activity of Thermoplastic Polyurethane Materials with Silver Nanoparticles

**DOI:** 10.3390/nano13091467

**Published:** 2023-04-25

**Authors:** Rocío Díaz-Puertas, Enrique Rodríguez-Cañas, Melissa Bello-Perez, Marta Fernández-Oliver, Ricardo Mallavia, Alberto Falco

**Affiliations:** 1 Institute of Research, Development and Innovation in Healthcare Biotechnology of Elche (IDiBE), Miguel Hernández University (UMH), 03202 Elche, Spain; r.diaz@umh.es (R.D.-P.); enrique.rodriguezc@umh.es (E.R.-C.); marta.fernandezo@umh.es (M.F.-O.); 2 Centro Nacional de Biotecnología (CNB-CSIC), Departamento de Biología Molecular y Celular, Campus de la Universidad Autónoma de Madrid, c/Darwin 3, 20849 Madrid, Spain; mloreto@cnb.csic.es

**Keywords:** 3D nanomaterials, thermoplastic polyurethane, ceramic, Ag nanoparticles, viricidal activity

## Abstract

The use of diverse Ag-based nanoparticulated forms has shown promising results in controlling viral propagation. In this study, a commercial nanomaterial consisting of ceramic-coated silver nanoparticles (AgNPs) was incorporated into thermoplastic polyurethane (TPU) plates using an industrial protocol, and the surface composition, ion-release dynamics and viricidal properties were studied. The surface characterization by FESEM-EDX revealed that the molar composition of the ceramic material was 5.5 P:3.3 Mg:Al and facilitated the identification of the embedded AgNPs (54.4 ± 24.9 nm). As determined by ICPMS, the release rates from the AgNP–TPU into aqueous solvents were 4 ppm/h for Ag and Al, and 28.4 ppm/h for Mg ions. Regarding the biological assays, the AgNP–TPU material did not induce significant cytotoxicity in the cell lines employed. Its viricidal activity was characterized, based on ISO 21702:2019, using the Spring viraemia of carp virus (SVCV), and then tested against the severe acute respiratory syndrome coronavirus 2 (SARS-CoV-2). The results demonstrated that AgNP–TPU materials exhibited significant (75%) and direct antiviral activity against SVCV virions in a time- and temperature-dependent manner. Similar inhibition levels were found against SARS-CoV-2. These findings show the potential of AgNP–TPU-based materials as a supporting strategy to control viral spread.

## 1. Introduction

Infectious diseases are a major global health concern and can have a significant negative socio-economic impact, particularly in developing countries. According to the World Health Organization (WHO), infectious diseases are a leading cause of morbidity and mortality, responsible for approximately 25% of all fatalities worldwide [1]. In particular, viruses can infect a wide range of hosts, including animals, plants and microorganisms [2]. In humans, they can cause multiple diseases with a wide ranging severity of prognoses from the common cold to more critical conditions such as acquired immune deficiency syndrome (AIDS) [3], Ebola virus disease (EVD) [4], Middle East respiratory syndrome (MERS) [5] or severe acute respiratory syndrome (SARS) [6]. In this sense, it is worth mentioning the recent global COVID-19 pandemic caused by SARS-CoV-2 and initiated in December 2019, which officially resulted in more than 250 million infected people and 5 million deaths within less than two years [7].

Viruses can be transmitted through various routes including direct or physical contact, oral (ingestion), sexual, vertical, vector-borne, aerosol (airborne) and fomites (contaminated inanimate objects) [8]. In the latter case, especially through frequently used objects in crowded and healthcare settings [9], fomites can be an important contributor to the spread of viruses such as human adenoviruses (HAdV) [10], rotavirus [11], human rhinovirus (hRV) [12], norovirus [13], influenza A virus (IFV-A) [14], SARS coronavirus [15], MERS-CoV [16] or SARS-CoV-2 [17].

The viability of the viral particles on fomites depends on the stability of their components and structural arrangements, which can be affected by environmental factors occurring over time such as humidity, temperature and irradiation [9]. For this reason, DNA and non-enveloped viruses are usually more resistant to adverse environments [18,19]. Associated with this, the chemical nature of the fomites also plays an important, sometimes crucial, role. For instance, SARS-CoV-2 shows less stability on porous materials (e.g., cotton) in comparison to non-porous surfaces (glass, plastic, metal, etc.), where infective virions were detected for 4 to 21 days [20].

In addition to new disinfection systems such as more sophisticated germicidal light irradiation treatments [21], new strategies to limit fomite-mediated virus transmission are focused on the development and implementation of new materials with upgraded viricidal properties [22]. Advances in the field of nanotechnology, such as nanomaterial coating strategies or nanoparticle (NP)-based antiviral agents, offer new approaches to prevent the spread of viruses [23]. NPs have emerged as antimicrobial materials due to their high surface-to-volume ratio and the specific chemical and physical properties with which they can be designed [24]. The use of metallic NPs as viricidal agents has grown rapidly due to their ability to target different molecular sites in viruses and, therefore, minimize resistance development events [25]. Two main mechanisms have been proposed to explain how metallic NPs, usually silver ones (AgNPs), interfere with viral replication: (i) via sulfur-bearing residues on cell and virus surface glycoproteins, preventing the attachment and entry of the virus into the host cell, and (ii) via cell membrane penetration and effectively blocking internal cellular factors necessary for the proper assembly of viral progeny [26]. Recently, the ability of AgNPs to inhibit SARS-CoV-2 has been investigated [27,28]. AgNPs (diameter: 2–15 nm) were capable of inhibiting extracellular SARS-CoV-2 virions and preventing their entry to host cells by disturbing viral integrity [27].

AgNP preparations in liquid environments limit their use for antiviral applications; however, great progress is being made in the use of AgNPs in solid and semi-solid preparations [29]. Recently, the incorporation of AgNPs into electrospun nanofibrous membranes of thermoplastic polyurethane (TPU) and polyvinyl alcohol (PVA) for the manufacture of personal protection equipment was studied, and their antiviral activity against Human immunodeficiency virus-1 (HIV-1) and SARS-CoV-2 was evaluated. Briefly, AgNP-loaded TPU (AgNP–TPU) nanofibers were found to be more effective in inhibiting HIV-1 and SARS-CoV-2 than those made of PVA [30]. In this sense, TPU’s interesting properties such as ease of processability, high durability, flexibility, excellent biocompatibility and great abrasion resistance make it suitable for a wide range of applications [31]. However, to the best of our knowledge, the use of TPU as a matrix scaffold for AgNPs for antimicrobial and, especially, antiviral applications has only been reported in the form of nanofibers.

The development of 2D and 3D AgNP–TPU nanomaterials as antimicrobial coatings for implementation in strategic areas to support public health could help slow the spread of some viral diseases. In this work, the viricidal ability of AgNP–TPU materials produced by an industrial protocol was assessed according to the general methodology described in the ISO 21702:2019 international standard, compliance with which is obligatory to certify this quality for plastics and other non-porous surfaces, but rarely addressed in scientific reports. Additionally, the surface and inner morphology of the materials were characterized, as well as their composition, including the release dynamics of metal ions in contacting aqueous solutions. Taken together, this study also aims to provide useful descriptions of methodological approaches to evaluate the potential implementation of experimental materials in virus protection applications.

## 2. Materials and Methods

### 2.1. Materials

Commercial additive 746-3BV (Esenttia, Bogotá, Colombia) consisting of AgNPs encapsulated in a polyethylene ceramic material (D98 < 40 μm) at 16% *w/w* in molten polypropylene (0.91 g/cm^3^, Duraflon^®^) was homogeneously mixed in a 1:16 ratio with molten thermoplastic polyurethane (TPU) (1.22 g/cm^3^, Ultimaker BV) at 220 °C to produce the AgNP–TPU material. Control TPU material was fabricated in the same way without adding the commercial AgNP additive. All products were supplied and assembled by Soorim 3D Solutions S.L. (Alicante, Spain), which is also the holder of the patent for the use of these materials for antimicrobial applications (patent number: U202031871).

### 2.2. Experimental Design

**Figure 1:**
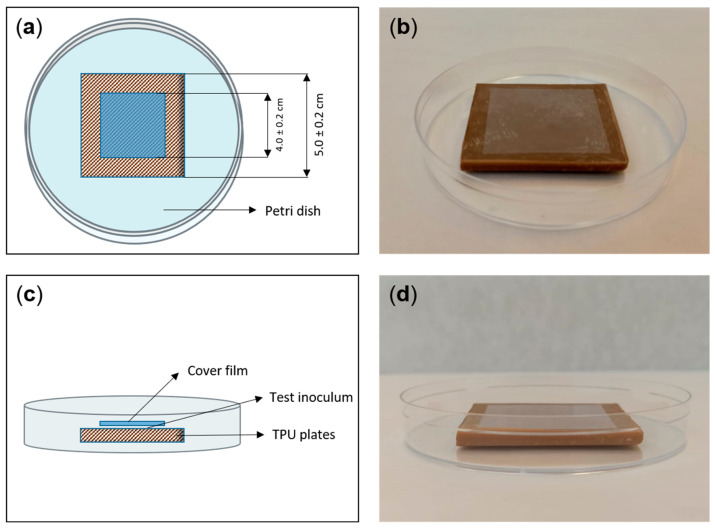
Schematic representations of the top (**a**) and side (**c**) views of the material and cover film layout, and corresponding example pictures ((**b**) and (**d**), respectively).

The experimental design of the analytical and biological trials was carried out by following the general guidelines described in ISO 21702:2019: “Measurement of antiviral activity on plastics and other non-porous surfaces”. Thus, the materials to be tested and their respective controls were molded to meet the dimensions required by this procedure (5 × 5 × 0.5 cm plates). Figure 1 shows schematic and visual representations of the disposition of the materials. Briefly, the AgNP–TPU and control TPU plates were placed in sterile Petri dishes and 400 µL of test inoculum was added onto the surface of the materials. Then, 4 × 4 cm polypropylene cover films were placed, covering and spreading the test inoculum towards the film edges without letting it leak beyond. The incubations were performed in such an arrangement inside humid chambers and at constant temperature. The temperature and duration of the incubations, as well as the type of test inoculum, varied depending on each particular test. All experiments were performed in triplicate.

### 2.3. Characterization of the Material Surface, the Released AgNPs and the Ion-Release Dynamics

The TPU plates were visualized using a Schottky-type Sigma 300 VP field emission scanning electron microscope (FESEM) with a coupled energy dispersive X-ray system (EDX) to determine their element composition (Carl Zeiss Microscopy GmbH, Oberkochen, Germany). Images were generated using both backscattered electron (BSE) and variable pressure secondary electron (VPSE) detectors.

The average hydrodynamic diameter (HDD) and the polydispersity index (PDI) of the AgNPs potentially released from the experimental materials into Milli-Q water inocula (co-incubated for 24 h at 20 °C) were determined by dynamic light scattering (DLS) using a 90 Plus Nanoparticle Size Analyzer (Brookhaven Instruments Corporation, Holtsville, NY, USA) [32]. All measurements were performed three times at 25 °C.

The ionic composition of the moieties released from the TPU plates was determined by inductively coupled plasma mass spectroscopy (ICPMS; ICPMS-2030, Shimadzu Company, Kyoto, Japan) calibrated with 107 Ag, 27 Al and 24, 25 and 26 Mg standards, being regression coefficient values (R^2^) equal or higher than 0.9996 for all calibration curves. The test inoculum used for this assay consisted of Milli-Q water samples incubated with the corresponding TPU plates at 20 °C for 2 and 8 h, as well as 24 h, which is the incubation time indicated by ISO 21702:2019 for testing the antiviral activity. Prior to analysis, these samples were diluted 1:30 (*v/v*) with nitric acid 1% (*v/v*), reaching a final volume of 3 mL. The non-diluted and non-treated water inocula, dried over silicon wafers, were also analyzed by FESEM-EDX (Carl Zeiss Microscopy GmbH) for this purpose.

### 2.4. Cells and Virus

The potential viricidal activity of the experimental TPU materials was tested with the fish pathogen Spring viremia of carp virus (SVCV, isolate 56/70) in epithelioma papulosum cyprinid cells (EPC; American Type Culture Collection ref.: CRL-2872) [33] and SARS-CoV-2 in Vero E6/TMPRSS2 cells [21].

EPC cell monolayers were grown at 28 °C in a 5% CO_2_ atmosphere in a Dutch modified Roswell Park Memorial Institute (RPMI) 1640 medium (Sigma, St. Louis, MO, USA) containing 10% fetal bovine serum (FBS; BioWhittaker, Inc., Walkersville, MD, USA), 1 mM sodium pyruvate (Sigma), 2 mM glutamine (Sigma), and 50 µg/mL gentamicin (Thermo Fisher, Waltham, MA, USA).

Vero E6/TMPRSS2 cell monolayers were grown at 37 °C in a 5% CO_2_ atmosphere in Dulbecco’s Modified Eagle Medium (DMEM, Sigma), supplemented with 10% FBS (BioWhittaker), 2 mM l-glutamine (Sigma), 1% non-essential amino acids (Sigma), and 100 units/mL penicillin and 100 µg/mL streptomycin (Thermo Fisher).

### 2.5. Cytotoxicity Assays

In compliance with ISO 21702:2019, prior to assessing the viricidal activity of the TPU materials, the potential citotoxicity of the moieties released from them when incubated with aqueous inocula was tested on EPC and Vero E6/TMPRSS2 cells. Thus, their corresponding culture media were used as test inocula and incubated at 20 °C (EPC medium) or 30 °C (Vero E6/TMPRSS2 medium) for 24 h, with both control and AgNP–TPU materials. Cell viability changes were determined by the reduction of 3-(4,5-dimethylthiazol-2-yl)-2,5-diphenyltetrazolium bromide tetrazolium (MTT, Panreac AppliChem, Barcelona, Spain) assay. Briefly, confluent cell monolayers in 96-well plates were treated with the collected inocula at 1:10 and 1:100 dilutions in corresponding fresh media (100 µL/well). After 24 h of incubation at each cell-type growing conditions, treatments were replaced by a cell medium containing 0.5 mg/mL MTT (from ten-fold concentrated stocks in phosphate-buffered saline (PBS, Gibco, Thermo Fisher)); in fresh media (100 µL/well) was used to replace treatments. MTT solutions were incubated with cells under the same conditions for 2 h and then carefully removed. The colored formazan product was dissolved in 100 µL of dimethyl sulfoxide (DMSO, Sigma) and the absorbance at 570 nm (and 620 nm as reference) was measured by means of a Cytation™ 3 cell imaging multi-mode microplate reader (BioTek Instruments, Inc., Winooski, VT, USA). Optical density is expressed in percentages relative to the control group consisting of untreated cells. Therefore, cell viability was calculated by the formula: 100× treated cell absorbance/untreated cell absorbance.

### 2.6. In Vitro Viral Infections

SVCV was used for the general characterization of the viricidal properties of the experimental TPU materials. Thus, SVCV inocula at 10^5^ focus-forming units (ffu) per mL in 2% FBS culture medium (SVCV infection medium) were incubated with the TPU plates, as described above, testing different temperatures (5, 10, and 20 °C for 24 h) and time periods (2, 8, and 24 h at 20 °C). Regarding SARS-CoV-2, inocula at 5 × 10^5^ plaque-forming units (pfu) per mL were tested at 30 °C for 24 h. For each condition, corresponding virus inocula were incubated with control TPU plates. Virus inactivation was determined as the viral titer reduction in percentage relative to control samples. SVCV and SARS-CoV-2 titers were determined by the focus-forming [33,34] and plaque assay [21] methods, respectively. All the experiments involving SARS-CoV-2 were performed in biosafety level 3 (BSL-3) facilities at CNB-CSIC according to the guidelines of the institution.

### 2.7. Statistical Analysis and Graphics

Data are shown as mean and standard deviation (SD) (n = 3, unless stated otherwise). Prism v7 (GraphPad software, La Jolla, CA, USA) and Microsoft Excel from the Microsoft Office Professional Plus 2019 package (Microsoft Corporation, Redmond, WA, USA) were used for creating the graphs. Prism v7 was used for performing the statistical analysis depending on each particular experimental design. Significant differences are indicated as: * (*p* < 0.05), ** (*p* < 0.01), and *** (*p* < 0.001).

## 3. Results and Discussion

### 3.1. Physicochemical Characterization of the Experimental TPU Materials

#### 3.1.1. Morphology and Composition

**Figure 2:**
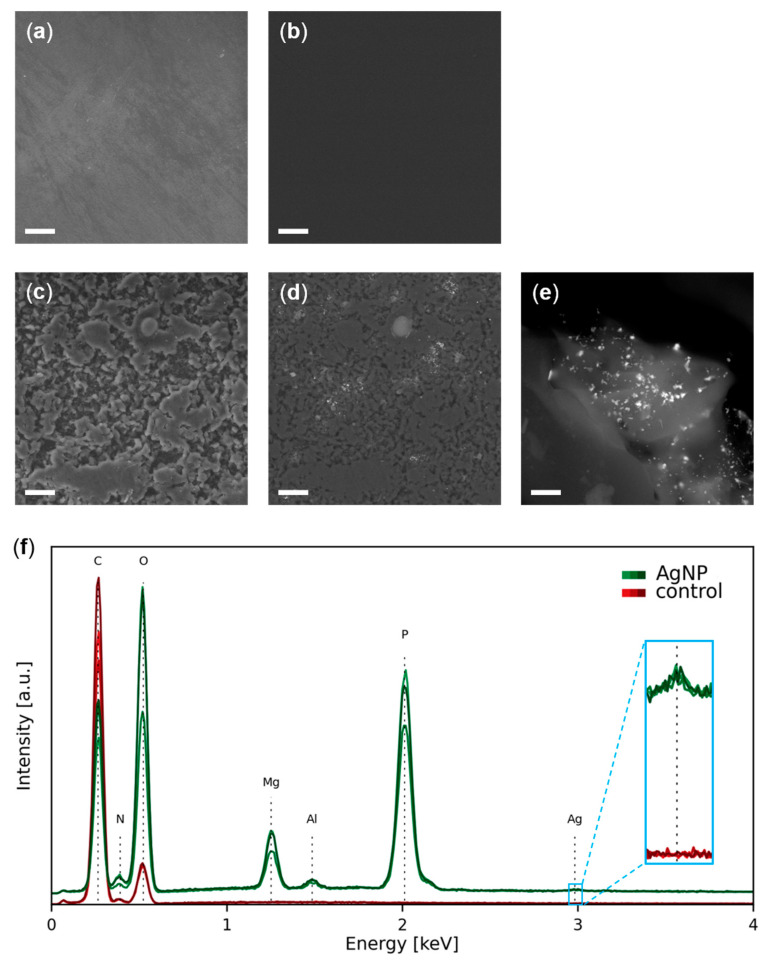
Representative FESEM images of the surface of the TPU (**a**,**b**) and AgNP–TPU (**c**–**e**) materials using VPSE (**a**,**c**) and BSE (**b**,**d**,**e**) detectors. The dark background in BSE images corresponds to the TPU moiety, the grey granules to the ceramic additive and the bright dots to the AgNPs. Scale bar of 20 µm for images (**a**–**d**) and 500 nm for image (**e**). (**f**) EDX analysis of the surface of the experimental materials. Three representative spectra for each type of material are shown. The analysis sites for the control material were randomly chosen, for the AgNP–TPU materials were restricted to the granulated regions. a.u., arbitrary units.

According to the FESEM images (Figure 2a–e) and the EDX analysis (Figure 2f) from the surface of the experimental materials, most of the elements are carbon and oxygen and correspond to the TPU moiety of both control TPU and AgNP–TPU materials, which is their matrix component and is visualized as dark background in the BSE images (Figure 2b,d,e). In these images, irregular light-grey granulation is also observed in the surface of the AgNP–TPU plates (Figure 2b,d), which belongs to the ceramic additive since it is associated to the presence of magnesium, aluminum and phosphorus, as determined by EDX spectroscopy (Figure 2f). Within such granule-like forms of 9.9 ± 6.4 µm (average value for 25 granules from different FESEM images), bright dots corresponding to AgNPs of 54.4 ± 24.9 nm (average value for 25 NPs from different granules) were also detected (Figure 2e,f).

**Figure 3:**
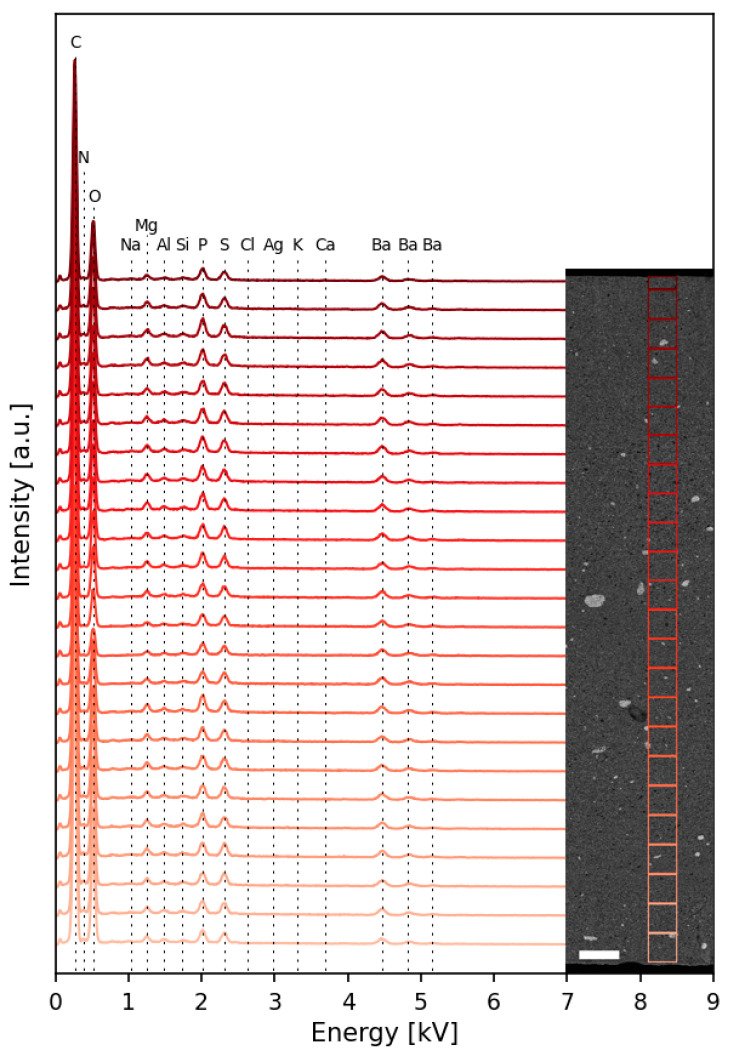
EDX analysis of the surface composition of the cross section of the AgNP–TPU material. a.u., arbitrary units. Scale bar: 200 µm.

It is worth mentioning that such a pattern is also shown along the entire cross section of the AgNP–TPU plates, as can be observed in the FESEM image from Figure 3. Several general EDX analyses across the surface of the section (Figure 3) confirm the composition described above, as well as the presence of traces of other elements, due to the much higher area analyzed (around 140 µm^2^) for each spectrum in comparison to the spectra shown in Figure 2 (punctual measurements).

The chemical composition of the components released from the AgNP–TPU material into contacting solutions was also studied by EDX spectroscopy. For this purpose, Milli-Q-water samples were incubated over the experimental materials, as described in the Material and Methods section herein and the ISO 21702:2019. Incubations were carried out at 20 °C for different time periods (2, 8 and 24 h). Thus, no differential major elements were found in the composition of the inocula incubated with control TPU plates in comparison to that of the original solution. In contrast, the ceramic components (P, Mg and Al) and Ag were detected in the inoculum samples from AgNP–TPU materials. It is worth highlighting the noteworthy non-release into the inocula (i.e., the absence) of those trace elements found in the general composition of the solid materials described in Figure 3: mainly S and Ba ions corresponding to barium sulphate molecules that are usually employed as filler additive in TPU materials.

**Figure 4:**
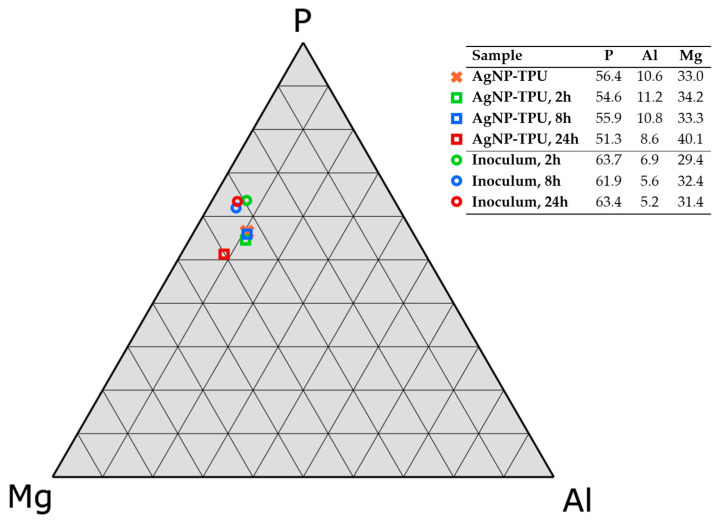
Ternary diagram of the relative populations of the ceramic components (P, Mg and Al) from the commercial additive in the experimental materials (square symbols) and the corresponding inocula (circular symbols) after incubation for 2, 8 and 24 h at 20 °C. Data obtained by EDX spectroscopy. Results shown as the mean value from three independent measures, relative error lower than 0.5%.

Figure 4 shows a ternary diagram displaying the relative amounts of P, Mg and Al ions detected in the surface of AgNP–TPU plates and the inocula at each time point. The ratio between the indicated components was found to be 5.5 P: 3.3 Mg: Al (table inset in Figure 4) or, in atomic composition, 4.8 P: 3.7 Mg: Al for the AgNP–TPU solid material. Similar percentages were found for the inocula, although with a notable increase in P, probably as a consequence of its higher partial solubility in aqueous solutions.

DLS analysis confirmed the absence of NPs in the inocula recovered from control TPU materials. On the contrary, they were detected in the inocula incubated with AgNP–TPU plates. Their mean HDD and PDI values were 1032 ± 148 nm and 0.382 ± 0.152, respectively (Figure S1). Regarding PDI values, they range from 0 to 1 and indicate the breadth of the particle size distribution. PDI values below 0.3 typically indicate a uniform distribution of particle sizes [35]. The values obtained are slightly higher than this threshold, which can be attributed to mild aggregation events that are common in metallic nanoparticles and depend on several chemical parameters of their immediate environment [36,37]. This may explain why particle size measurements obtained by this technique are much larger than expected.

#### 3.1.2. Ceramic Component Degradation Dynamics and Ion Release Rate

**Figure 5:**
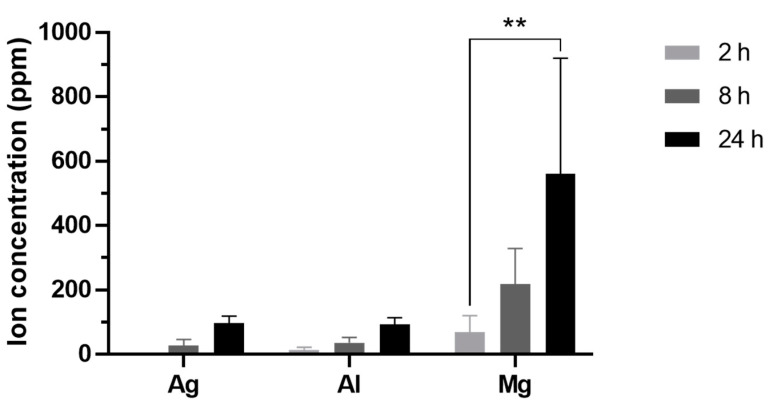
Time evolution of the concentration of Ag, Al and Mg (ppm) cumulated in the aqueous inocula released from AgNP–TPU materials after their incubation for 2, 8 and 24 h at 20 °C. Analysis performed by ICPMS. The results are represented as means ± SD (n = 3). Statistically significant differences between groups (two-way ANOVA with Tukey’s multiple comparisons correction) are shown on binding braces. **, *p* < 0.01.

The same water inocula incubated with the materials and previously characterized by EDX were also analyzed by ICPMS for a more precise quantitation of relevant components released from the nanoparticles (Ag) and the ceramic matrix (Al and Mg) of the AgNP–TPU materials. According to these data (Figure 5), there is a significant increment of ions in the co-incubated inocula along time (F = 7.234; *p* < 0.01), as well as a significant difference of cumulated ions between the elements studied (F = 10.45; *p* < 0.001). In these regards, there are several conditioning factors such as, mainly, the ability of each element to be released from the source material and their solubility in water. In comparison to other studies, similar delivery trends associated with the degradation of ceramic components have already been described [38,39]. In quantitative terms for each particular element, the release rate of Ag and Al ions was approximately 4 ppm/h for each one, while that of Mg was about six times higher (28.4 ± 5.7 ppm/h).

### 3.2. Biological Properties

#### 3.2.1. Cytotoxicity Assays

**Figure 6:**
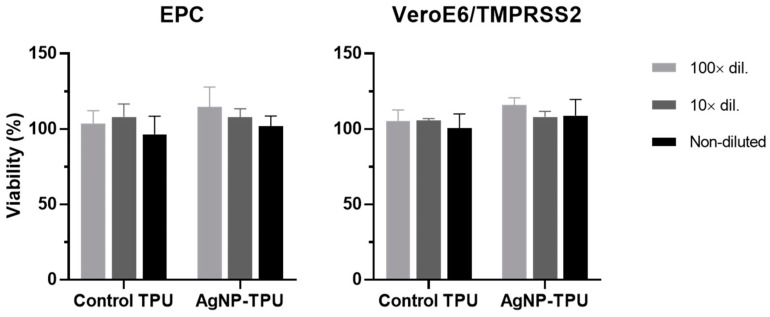
Cytotoxicity in EPC and VeroE6/TMPRSS2 cells of culture media previously incubated with the experimental materials. The inocula were tested undiluted, 1:10 and 1:100 diluted in cells. The results correspond to four independent experiments and are represented as means with SD in viability percentages in comparison to the viability obtained from the treatment of cells with culture medium incubated independently with no contact of any of the materials. Statistically significant differences between groups were calculated by using two-way ANOVA with Tukey’s multiple comparisons correction; however, no statistically significant differences were found.

The potential cytotoxicity of the moieties released from the experimental materials was assessed before determining their viricidal activity. For this purpose, EPC and Vero E6/TMPRSS2 media were incubated with the TPU plates at 20 °C and 30 °C, respectively, for 24 h. Then, they were used at different dilutions to treat the corresponding cells for 24 h before performing the MTT assay. Thus, as shown in Figure 6, no significant viability changes were observed in any of the cases (F = 0.873 and *p* = 0.4929 for EPC; and F = 0.9511 and *p* = 0.4576 for Vero E6/TMPRSS2).

Although this aspect has been extensively studied for AgNPs in different cell lines, there is no consensus on their particular cytotoxic concentration since this is affected by several factors such as particle size, medium type, temperature, surface functionalization and particle crystallinity [40]. Based on our ICPMS analysis, the Ag concentration in the non-diluted inocula was about 100 ppm (i.e., 100 µg/mL) after 24 h of incubation with the experimental materials. Therefore, these cytotoxicity results are consistent with previous studies showing that AgNPs at a concentration of up to 100 μg/mL do not decrease the viability of progenitor human adipose-derived stem cells [41]. Neither did Carrola et al. (2016) observe cytotoxicity in HaCaT keratinocytes after 48 h of treatment with Ag nanoprisms and spherical AgNPs at a concentration of 100 ppm [42], or Rogers et al. (2008) with 100 ppm of 10-nm AgNPs in Vero cells [43]. Nevertheless, other authors did observe cytotoxicity at equal or lower concentrations in HEK293 [44], HT22 [45], Calu-3 and Vero E6/TMPRSS2 [27] cells.

It is important to note here that the mechanisms underlying the toxicity of AgNPs, which are also largely related to their antimicrobial activity, operate at different molecular and cellular levels and are still under investigation. Therefore, although these nanomaterials are already present in a wide range of consumer products; due to their potential impact on the environment and public health; further studies are needed in this regard to ensure their safety, in particular for environmental, health, food and textile applications [46].

#### 3.2.2. Viricidal Activity

**Figure 7:**
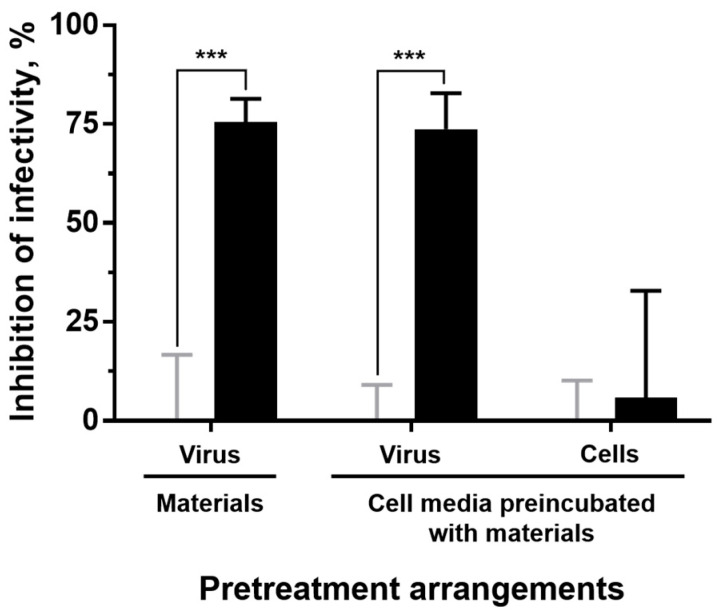
Potency and origin of the antiviral activity against SVCV of the experimental materials. To assess the type of antiviral activity exerted by the materials, three different pretreatment arrangements were performed (in order): direct incubation of the virus inoculum with the material; and the treatment (prior to infection) of either virus stocks or host cells with 2% FBS culture medium that had been previously incubated with the materials. The results correspond to three independent experiments and are represented as means ± SD of the infectivity inhibition associated with AgNP–TPU materials (black bars) in percentages relative to corresponding control groups (i.e., viricidal activity of control TPU materials, grey bars). Statistically significant differences between corresponding control and AgNP–TPU groups (two-way ANOVA with Tukey’s multiple comparisons correction) are shown: ***, *p* < 0.001.

Once it was verified that the inocula previously incubated with the experimental materials did not exert relevant cytotoxic effects on the host cells, we proceeded to determine whether they were endowed with viricidal properties and, if so, the specific source of such activity within the material (Figure 7). Thus, SVCV inocula showed an about 75% titter reduction when directly incubated with the AgNP–TPU plates in comparison to the titters determined for the incubations with the control material. We then revealed that such antiviral activity comes from the moieties of the material that are released into the inoculum during the incubations; since when they consist of just medium and are used to treat SVCV, virus infectivity is inhibited to the same extent as in SVCV-material direct incubations. Finally, it was also demonstrated that this effect is not due to a reduction in the sensitivity of cells to viral infections, since the treatment of host cells with culture medium that had been previously incubated with the materials did not confer antiviral protection on them. We note that all incubations and treatments were performed at 20 °C for 24 h.

**Figure 8:**
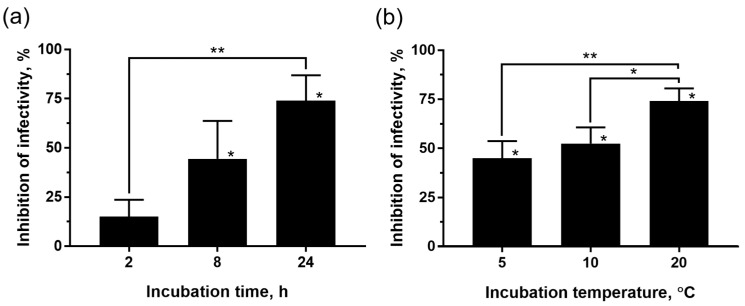
Effect of (**a**) the incubation time (2, 8 and 24 h) at 20 °C and (**b**) the incubation temperature (5, 10 and 20 °C) for 24 h, in the viricidal activity against SVCV of the AgNP–TPU materials. The results correspond to three independent experiments and are represented as means ± SD of the infectivity inhibition in percentages relative to corresponding control groups. Statistically significant differences between groups (one-way ANOVA with Tukey’s multiple comparisons correction) are shown on binding braces. Statistically significant differences compared to their respective control groups (two-tailed unpaired Student’s t-test with Welch’s correction) are shown above each column. *, *p* < 0.05; **, *p* < 0.01.

To further characterize the viricidal activity of the materials, different incubation periods (24 h as stated in the ISO 21702:2019, but also 2 and 8 h) at 20 °C were tested. The results obtained (Figure 8a) indicate that the incubation time has an effect in this regard by reducing the virus infectivity along time (one-way ANOVA, F = 12.87, *p* < 0.01). The inhibition of the infectivity was significant in virus inocula incubated for 8 (44.4 ± 19.3%) and 24 h (74.1 ± 12.8%) with the AgNP–TPU plates, in comparison to control groups (Student’s t-test, *p* < 0.05). In addition, such time-dependent inhibition of virus infectivity coincides with the Ag release trend shown before for the AgNP–TPU materials (Figure 5).

Several studies have reported the antiviral activity of AgNPs against a range of viruses such as herpes simplex virus (HSV) [47], HIV [48], IFV-A [49], monkeypox virus (MPV) [43], Newcastle disease virus (NDV) [50], respiratory syncytial virus (RSV) [26] or SARS-CoV-2 [27]. Particularly, in the study of Rogers et al. (2008), a concentration of 100 ppm of 10-nm AgNPs decreased the plaque formation of the MPV by 79% in Vero cells [43]. In a recent study, inhibitions of 64 to 79% in the replication of NDV using AgNPs at concentrations of 80 to 320 ppm [50] were also shown. Altogether, these results are consistent with the data obtained in this study since the exposure of the inocula to the AgNP–TPU material for 24 h delivered Ag ions up to a concentration of around 100 ppm into them, thus conferring in them the ability to inhibit SVCV infectivity by up to about 74%.

Similarly, the viricidal activity of the AgNP–TPU materials was also found to depend on the temperature of incubation (Figure 8b, one-way ANOVA, F = 11.16, *p* < 0.01). For these assays, incubation temperatures of 5 and 10 °C were tested, in addition to 20 °C. The exposure time was set at 24 h, as specified by ISO 21702:2019. Ultimately, incubation with the AgNP–TPU material reduced SVCV infectivity by 45 ± 8.7% at 5 °C, 52.4 ± 8.2% at 10 °C, and 74.1 ± 6.4% at 20 °C. These results are in line with previous studies describing that the dissolution of AgNPs in an aqueous solution increases with temperature [51,52]. The temperature also affects the release profile of several metallic ions from nanoparticles in the same way, due to the temperature dependence of the ionic diffusion coefficient. Indeed, Zhang et al. (2011) developed a kinetic model using the Arrhenius equation (Equation (S1)) to explain the release of Ag ions based on the hard sphere theory [53]. According to this equation, the higher the temperature, the higher the release rate of Ag ions.

Finally, the viricidal activity of the experimental materials was also tested against a clinically relevant virus, SARS-CoV-2. For these assays, the virus inocula were incubated with the TPU materials at 30 °C for 24 h prior to their titration. According to the data, the AgNP–TPU plates reduced the SARS-CoV-2 infectivity in 80.77 ± 14.73% in comparison to the TPU control (two-tailed Mann-Whitney *U* test, n = 4, *p* = 0.0286), and, therefore, this was similar to the inhibition ranges determined against SVCV.

## 4. Conclusions

This study aimed to evaluate the viricidal ability (by following the general guidelines of ISO 21702:2019) against SVCV (virus model) and SARS-CoV-2 of a composite material consisting of a TPU matrix with embedded AgNPs. Thus, this material exerts a viricidal activity of about 75% directly through the AgNPs released into the inoculum with which it is incubated. In addition, such activity is shown to be time- and temperature-dependent, probably due to the release trend over time of Ag ions from the AgNP-materials shown here. The compounds released by the AgNP–TPU materials do not present significant levels of cytotoxicity in EPC and Vero E6/TMPRSS2 cells under the tested conditions. These findings postulate TPU–AgNP as promising candidate materials for viricidal applications to decrease the spread of viruses such as SARS-CoV-2.

## Data Availability

The data presented in this study are available on request from the corresponding author.

## Supplementary Materials

The following supporting information can be downloaded at: https://www.mdpi.com/article/10.3390/nano13091467/s1, Figure S1. Hydrodynamic diameter (HDD) determined by dynamic light scattering (DLS) of NPs released into Milli-Q water inocula incubated with AgNP-TPU materials for 24 h at 20 °C.
