# Supplementary material for: Viricidal Activity of Thermoplastic Polyurethane Materials with Silver Nanoparticles
Source: Nanomaterials (Basel). 2023 Apr 25;13(9):1467. doi: 10.3390/nano13091467 (PMC10180066; doi:10.3390/nano13091467)
Supplement: Supplementary file 1 [file nanomaterials-13-01467-s001.zip › nanomaterials-2298639-supplementary.pdf]

nanomaterials MDPI

Supplementary Material

Viricidal Activity of Thermoplastic Polyurethane Materials with Silver Nanoparticles

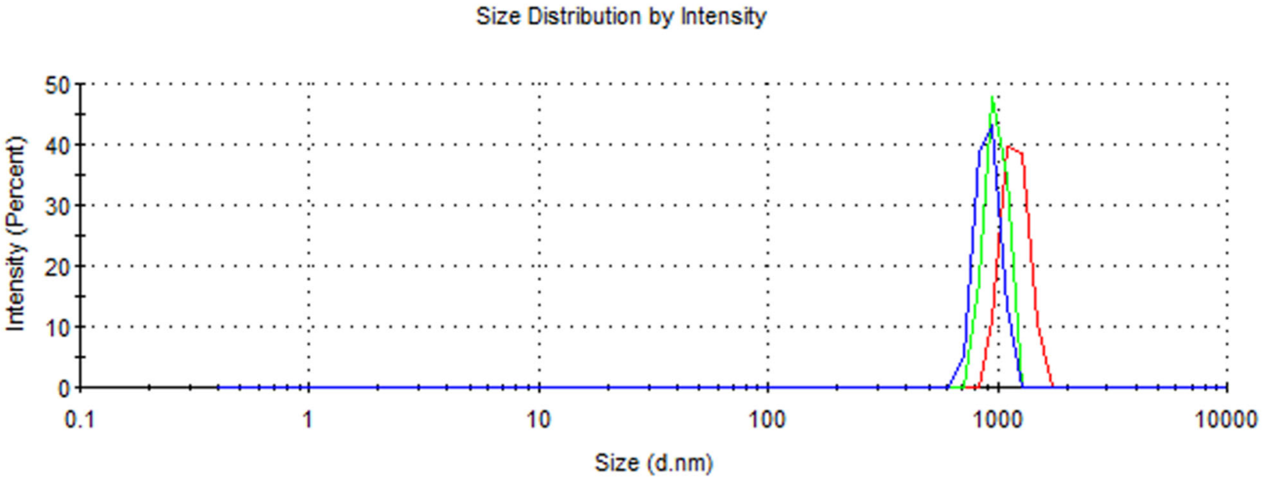

Figure S1. Hydrodynamic diameter (HDD) determined by dynamic light scattering (DLS) of NPs released into Milli-Q water inocula incubated with AgNP-TPU materials for 24 h at 20 °C. Results from three independent experiments, each shown in a different color.

$$\gamma_{Ag^+} = \frac{3}{4}\left(\frac{8\pi k_b T}{m_B}\right) p^{-1} exp\left(\frac{-E_a}{k_b T}\right) [Ag]_r{}^{-1} [O_2]^{0.5} [H^+]^2 \quad \text{(S1)}$$

Equation S1: Kinetic model based on the hard sphere theory using the Arrhenius equation. γ_{Ag^+}: Ag^+ release rate; k_B: Boltzmann constant (1.38x10^{-23} J/K); T: temperature (298K); m_B: molecular weight of oxygen or protons; E_a: activation energy (J); $[Ag]_r$: released Ag^+ concentration. Developed by Zhang et al. (2011) [1].

References

1. Zhang, W.; Yao, Y.; Sullivan, N.; Chen, Y. Modeling the Primary Size Effects of Citrate-Coated Silver Nanoparticles on Their Ion Release Kinetics. *Environ. Sci. Technol.* **2011**, *45*, 4422–4428. https://doi.org/10.1021/es104205a.
